# Traumatic Brain Injury in Patients under Anticoagulant Therapy: Review of Management in Emergency Department

**DOI:** 10.3390/jcm13133669

**Published:** 2024-06-24

**Authors:** Vincenzo G. Menditto, Giulia Rossetti, Mattia Sampaolesi, Marta Buzzo, Giovanni Pomponio

**Affiliations:** 1 Emergency and Internal Medicine Department, Azienda Ospedaliero Universitaria delle Marche, 60126 Ancona, Italy; mattia.sampaolesi@ospedaliriuniti.marche.it (M.S.); marta.buzzo@ospedaliriuniti.marche.it (M.B.); 2 Internal Medicine, Santa Croce Hospital AST1 Pesaro Urbino, 61032 Fano, Italy; giulia.rossetti1@sanita.marche.it; 3 Clinica Medica, Azienda Ospedaliero Universitaria delle Marche, 60126 Ancona, Italy; giovanni.pomponio@ospedaliriuniti.marche.it

**Keywords:** traumatic brain injury, oral anticoagulant, biomarkers, neurosurgery, intracranial injury

## Abstract

The best management of patients who suffer from traumatic brain injury (TBI) while on oral anticoagulants is one of the most disputed problems of emergency services. Indeed, guidelines, clinical decision rules, and observational studies addressing this topic are scarce and conflicting. Moreover, relevant issues such as the specific treatment (and even definition) of mild TBI, rate of delayed intracranial injury, indications for neurosurgery, and anticoagulant modulation are largely empiric. We reviewed the most recent evidence on these topics and explored other clinically relevant aspects, such as the promising role of dosing brain biomarkers, the strategies to assess the extent of anticoagulation, and the indications of reversals and tranexamic acid administration, in cases of mild TBI or as a bridge to neurosurgery. The appropriate timing of anticoagulant resumption was also discussed. Finally, we obtained an insight into the economic burden of TBI in patients on oral anticoagulants, and future directions on the management of this subpopulation of TBI patients were proposed. In this article, at the end of each section, a “take home message” is stated.

## 1. Background

### 1.1. Definition and Epidemiology

Traumatic brain injury (TBI) is the alteration in the normal function of the brain provoked by crash, blow, jerk to the head, or penetrating injury [1]. It differs in severity from mild TBI (mTBI), accounting for 80% of cases, to moderate TBI (moTBI) and severe TBI (sTBI) [2]. The economic and social burden of TBI is substantial and multifaceted, encompassing both direct and indirect costs, approximately estimated to be around USD 400 billion annually [3], which impact individuals, families, and society at large. Moreover, individuals with TBI frequently require immediate diagnostic workouts and treatment in emergency departments (EDs). 

Managing TBI in patients who are anticoagulated, principally for the prevention of ischemic stroke in atrial fibrillation and for the treatment of acute venous thromboembolism, presents unique challenges and requires careful consideration to balance the risks of bleeding with the risk of thrombosis. The high prevalence (up to 2.4%) of these patients among the adult population makes it of great relevance to public health and the routine activities of emergency professionals [4]. The proportion of patients with TBI on oral anticoagulation (OAT) is steadily increasing and is now estimated to be up to 38% [4]. The prevalence of intracranial injury in this cohort has been reported to be between 5 and 20% [5]. For many years, vitamin K antagonists (VKAs) were the most widely used OAT, but, now, direct oral anticoagulants (DOAs)—apixaban, edoxaban, rivaroxaban, and dabigatran—are largely prescribed [4].

*Take home message:* The most effective and safest management of patients who suffer from TBI while on OAT is one of the major concerns of emergency services. This is due to the increase in TBI incidence in the aging population and because the use of OAT is steadily increasing.

### 1.2. The Special Case of mTBI

There is a significant discrepancy in the literature regarding the definition of mTBI [6]. In a systematic review (SR), Carroll et al. [7] counted up to 38 different definitions for mTBI. Currently, the most widely accepted definition is that proposed by the World Health Organization Collaborating Centre Task Force on Mild Traumatic Brain Injury [8,9]. All following criteria must be fulfilled to match a diagnosis of mTBI: a Glasgow coma scale (GCS) score between 13 and 15 at 30 min after the injury plus one or more of the following symptoms: loss of consciousness < 30 min; post-traumatic amnesia (PTA) < 24 h; and impaired mental state at time of accident (confusion, disorientation, transient neurological deficit). However, critical issues seem to affect each point. For example, patients with a GCS of 13 had a higher incidence of intracranial injuries (ICIs) requiring surgical intervention, so these cases should be more consistent with those classified as moTBI [10,11]. Consequently, many authors excluded patients with a GCS of 13 from clinical studies about mTBI [12,13]. In addition to this, GCS is less reliable and easy to calculate in patients with pre-existing neurological disorders [14]. Moreover, the above-mentioned criteria allow for wide variability in the severity of mTBI in, for example, cases of minor, short-lived symptoms or loss of consciousness lasting up to 30 min [6]. As an additional element of confusion, there is a debate regarding whether the term “concussion” should be used as a synonym for mTBI, as stated in a 2023 American College of Emergency Physicians (ACEP) policy [15], or whether it should indicate a more severe form [16]. Other experts [17] prefer to reserve the term “concussion” for those cases with evidence of ICI on conventional neuroimaging or persistent neurological deficit. A universally accepted definition is needed, especially in the case of mTBI occurring in patients on OAT [18], when management and treatment options differ significantly.

*Take home message*: People with TBI are at high risk of ICI, and a GCS < 14 seems to be the most predictive clinical factor. Thus, we suggest classifying as affected by mTBI only those patients with a GCS of 14–15, paying particular attention to the assessment of subjects with pre-existing neurological disorders. The term “concussion” is an unnecessary source of confusion and should be forsaken.

## 2. Literature Search Strategy

A literature search in MEDLINE (PubMed), Google Scholar, and National Institute for Health and Clinical Excellence (NICE) databases was performed. We conducted an extensive literature search and a manual search (last updated May 2024), combining MeSH and free terms: traumatic brain injury; brain injury; and anticoagulants (Supplementary Figure S1). 

## 3. Diagnosis and Risk Stratification

### 3.1. Guidelines

The main guidelines (GLs) recommend the performance of a head computed tomography (CT) scan in all OAT patients [19,20,21]. However, these recommendations are not supported by solid evidence because of the scarcity of relevant studies of high methodological quality, such as randomized controlled trials (RCTs). As a result, all authors put a strong emphasis on expert opinions and clinical experience. In the documented GLs of the European Federation of the Neurological Societies (EFNS) [19] (GL3), OAT is a definite risk factor for ICI after mTBI; thus, a head CT scan is indicated for all anticoagulated patients and, in the case of a normal CT, observation of 24 h, consulting a neurotrauma center, and repeating the CT (or magnetic resonance imaging) are considered reasonable options. Also, Scandinavian GLs [20] suggest a head CT scan for all patients on OAT, even those with a GCS = 15, together with 24 h observation; repeating the CT scan is suggested in the case of neurological and/or GCS (≥2 points) deterioration. Recently, the 2023 NICE GLs [21] suggested considering conducting a head CT scan for people who have sustained a TBI and have no other indications for a head CT scan, except being on OAT. After a negative scan, these patients could be discharged safely, if no additional risk factors, such as the presence of other injuries, no supervision at home, high energy traumas, and high risk of further falls, emerge from a careful evaluation. Moreover, the panel agreed that the evidence was not strong enough to sustain a “do no admit solely based on anticoagulation status” recommendation. The Brain Injury GL (BIG) [22] defines which patients, after a positive head CT scan, require a period of observation, repeated head CT scan, or neurosurgical consultation based on patient history, physical examination, and initial head CT findings. In this GL, patients on warfarin are allocated to the most severe tertile (BIG3), which indicates the need for a complete diagnostic plan. 

The heterogeneity of TBI patients taking anticoagulants contributes to making the design of clinical research more complicated and the results hard to interpret. To date, only a few studies have focused specifically on recommendations regarding TBI patients on anticoagulants. An interdisciplinary group of Austrian experts [23] developed recommendations regarding the management of TBI in patients on anticoagulants. They stated that all patients with suspected or known TBI while on OAT require a CT scan regardless of anamnesis or neurological findings. Recently, Gallagher et al. [24] proposed a modified BIG for TBI patients on pre-injury anticoagulation with a positive head CT scan. They removed the “being on OAT” criterion from the protocol and re-stratified patients; in this way, the utilization of neurosurgical consultation could be decreased by up to 52%.

*Take home message*: In our experience, the EFNS GL seems to be the most reliable guide for the care of anticoagulated patients suffering from TBI. Indeed, in our opinion, the 2023 NICE, even though characterized by a more robust methodology and higher levels of evidence, does not place enough attention on the subgroup of anticoagulated patients.

### 3.2. Clinical Decision Rules

Risk factors for poor prognoses in TBI have been extensively studied [25,26], and many of them have been incorporated into clinical decision rules (CDRs) [25,27,28,29,30], which, undoubtedly, are tools of outstanding importance for clinicians practicing in any ED, particularly in the case of mTBI. For example, CDRs assist the clinician in identifying patients who have essentially no risk of significant ICI after mTBI and for whom a CT scan is therefore unnecessary [25,31].

Unfortunately, no specific decision rule dedicated only to OAT patients with mTBI is currently available. Despite OAT being considered a risk factor for bleeding complications by several CDRs, such as National Emergency X-Radiography Utilization Study (NEXUS) II [28,29] or the CT in Head Injury Patients (CHIP) prediction rule [32], in the two main validated CDRs—the Canadian CT Head Rule (CCHR) and the New Orleans Criteria (NOC) [30]—OAT was an exclusion criterion [31]. 

Recent evidence seems to suggest that the same clinical risk factors extensively studied in mTBI could be used in the first assessment of the subgroup of patients on OAT [33,34,35,36,37,38,39,40,41,42]. In a prospective study, Cipriano et al. [33] found that PTA and trauma above the clavicles remain independent predictors for ICI in people on OAT. Nonetheless, in patients on DOAs, only evidence of trauma above clavicles independently predicts ICI. Since 2019, most studies have focused on patients on DOAs. Some data [34,35,36], indeed, showed a lower risk of ICI in patients on DOAs compared to those on VKAs, justifying the introduction of specific management strategies. In two different analyses [36,37], Turcato et al. reported that major dynamics, PTA, post-traumatic transitory loss of consciousness (TLOC), GCS score < 15, post-traumatic headache, and evidence of trauma above the clavicles were associated with a higher likelihood of ICI in patients on DOAs. The same group [38] conducted a decision tree analysis with the chi-square automatic interaction detection (CHAID) method, a statistical machine learning technique, to analyze the relative weight of clinical risk factors in predicting the risk of ICI in patients taking DOAs. Two out of the six above-mentioned factors (GCS < 15 and post-traumatic headache) were excluded by the model and previous neurosurgery emerged as the strongest predictor. However, both studies were retrospective and there was a lack of a specific analysis about the risk of delayed ICI. Moreover, Fuller et al. [39] suggested that the absence of all clinical risk factors significantly reduces the risk of ICI in patients on DOAs. In 2023, Park et al. [40] confirmed the risk factors previously cited (the six criteria plus previous neurosurgery), adding post-traumatic vomiting (OR between 2.73 and 7.40). The authors proposed the HERO-M (Hemorrhage Estimate Risk in Oral Anticoagulation for Mild Head Trauma) nomogram, obtained by the sum of the individual weighted scores for each of the eight factors. This tool had a good ability to predict the probability of post-traumatic ICI (area under the curve [AUC]: 0.803; 95% CI: 0.721–0.884). However, because of the retrospective design of the study and the lack of a sample calculation and follow-up CT scan, further validation appears mandatory.

Recently, in a retrospective study, Turcato et al. [41] proposed a decision tree analysis using the “classification and regression tree” (CART) method, a new machine learning technique to analyze clinical risk factors for ICI in anticoagulated patients (VKAs and DOAs). The progressive exclusion of five risk factors—PTA, post-traumatic TLOC, greater trauma dynamic, GCS < 15, and evidence of trauma above the clavicles—reduced the risk of ICI from 61.4% to 2.5%. Finally, in a recent prospective study [42], we found that only post-traumatic severe headache (OR: 5.10; 95% CI: 1.26–20.75; *p* = 0.02) and post-traumatic vomiting (OR: 3.44; 95% CI: 1.20–9.89; *p* = 0.02) correlated with ICI on the first or second head CT scan.

*Take home message*: Many studies have evaluated mTBI in anticoagulated patients, but they were retrospective and the overall quality of the body of evidence was low as a result of imprecision, indirectness, and a high risk of bias. At present, a prospective study is currently in progress to determine whether the CCHR GLs could be applied to patients on OAT.

### 3.3. Role of Biomarkers

In acute TBI, biomarkers have been proposed to (a) grade the severity of brain damage, (b) predict prognosis, (c) guide clinical management, and (d) monitor therapeutic interventions. Candidate molecules include markers of neuronal cell damage, such as neuron-specific enolase (NSE) and ubiquitin carboxyl-terminal hydrolase isozyme L1 (UCHL1), of axonal damage, such as tau and neurofilament light (NFL), or of astrocyte damage, such as S100 calcium-binding protein B (S100B) and glial fibrillary acidic protein (GFAP) [43,44].

In patients suffering from mTBI, data indicate that biomarker serum levels could be effective in predicting the absence of ICI on head CT scans, reducing the need for CT examination and saving costs [45,46]. In 2018, serum measurement of GFAP, in combination with UCH-L1 (Abbott’s Duoset) was cleared by the Food and Drug Administration (FDA) for clinical use, aiming to identify patients with a higher likelihood of ICI on head CT scans within 12 h from trauma [45]. Many studies also tested S100B, GFAP, and UCH-L1 in this setting. However, only a few studies considered OAT an inclusion criterion [47,48,49], and no patients on OAT were included. In other studies [50,51], anticoagulated patients were included, but there was no analysis of biomarkers in this subgroup. 

Moreover, dosage of S100B was incorporated into the Scandinavian Neurotrauma GL [20] and, more recently, the testing of S100B, UCH-L1, and GFAP was proposed, with a strong agreement among experts, by the French Society of Emergency Medicine [52], again aiming to limit the request for head CT scans. However, neither in the Scandinavian GL nor in the French consensus was the dosage of biomarkers routinely recommended for anticoagulated patients. More recently, a document by the Spanish Society of Emergency Medicine [53] recommended the dosage of GFAP and UCH-L1 within 12 h in mTBI patients with GCS = 15 and the presence of one or more risk factors, such as ongoing OAT. To date, there are only two published studies [54,55] on the role of dosing S100B, NSE, GFAP, and UCH-L1 serum levels in anticoagulated patients with mTBI. David et al. [54] described the results of dosing S100B in 308 elderly patients on antithrombotic medication (30% were taking OAT). A negative predictive value (NPV) of 94.3% and a negative predictive value (PPV) of 12.7% for the diagnosis of ICI at the first head CT scan were reported. Recently, our group compared, for the first time, the performances of four biomarkers—S100B, NSE, GFAP, UCHL-1—and Alinity TBI (Abbott’s Duoset) after mTBI in anticoagulated patients [55]. The most important finding from our data was a 100% NPV of GFAP and Alinity TBI for the diagnosis of delayed ICI (dICI), using the cut-off values specified by the manufacturers. Moreover, we found a superiority of GFAP for discriminating CT-positive from CT-negative at the first head CT scan (NPV of 95.8%). Our data, mainly limited by a low number of events, if adequately confirmed in future studies, might suggest the utility of GFAP, using the hypothetical cut-off of 67 pg/mL, to reduce the need to repeat CT scans by approximately 40%. Moreover, the serum levels and performances of S100B, GFAP, and UCH-L1 seem to be similar in cases of VKAs or DOAs [55]. On the other hand, decrements in specificity and increased serum values of the biomarkers in elderly patients suggest that special attention should be paid to these patients [56]. It is noteworthy that the time, up to a couple of hours, necessary to obtain biomarker results could lead to a significant delay in organizing head CT scans.

In mo/sTBI, blood biomarkers have been studied to improve clinical assessment and prognostication. To date, there are no published studies on the role of dosing NSE, GFAP, or UCH-L1 serum levels in a subpopulation of anticoagulated patients. In a prospective study, Korhonen et al. [57] found in 85 patients with mo/sTBI that very high levels of GFAP and S100B seemed to be associated with poor prognosis and mortality. However, extracranial injuries, the timing of sampling, and demographic factors such as age and pre-existing systemic or neurological conditions could also play a significant role. In the work of Richter et al. [58], the serum biomarkers GFAP, NFL, S100B, and UCH-L1 improved outcome prediction, defined through the severity of imaging, after mo/sTBI, especially in patients with a Marshall score < 3. In the Collaborative European NeuroTrauma Effectiveness Research in Traumatic Brain Injury (CENTER-TBI) Core Study, six biomarkers were analyzed in 2867 patients (37.7% mo/sTBI), and all of them scaled with injury severity, classified according to the GCS, and care path intensity; GFAP was the best predictor for CT positivity [59]. In those cohorts, it was not specified if patients on AOT were included. Yuguero et al. [60] studied 540 patients with moTBI and found that within 6 h after TBI, high levels of S100B, but not of NSE, UCHL1, or GFAP, correlated with the development of complications. However, the authors did not provide an analysis of the biomarkers in this specific subgroup of patients on OAT (40% of the population).

**Figure 1:**
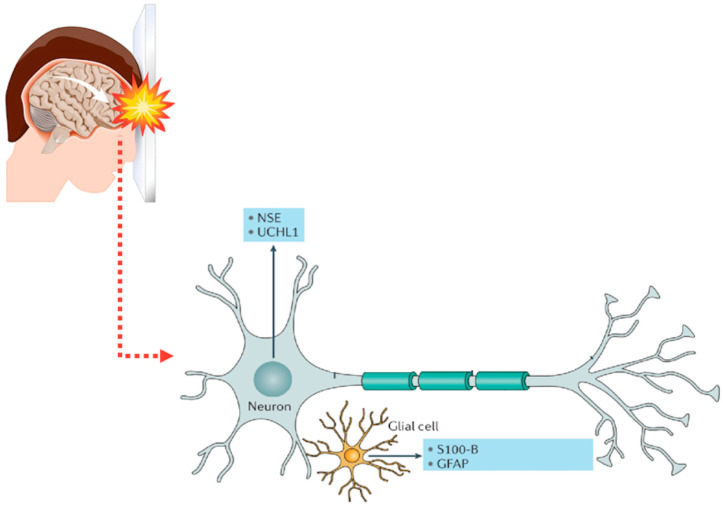
Some of the most studied brain biomarkers have been proposed as diagnostic and prognostic markers of traumatic brain injury. Modified from ref. [50]. S100B: calcium-binding protein B; NSE: neuron-specific enolase; UCHL1: ubiquitin carboxyl-terminal hydrolase isozyme L1; GFAP: glial fibrillary acidic protein.

**Figure 2:**
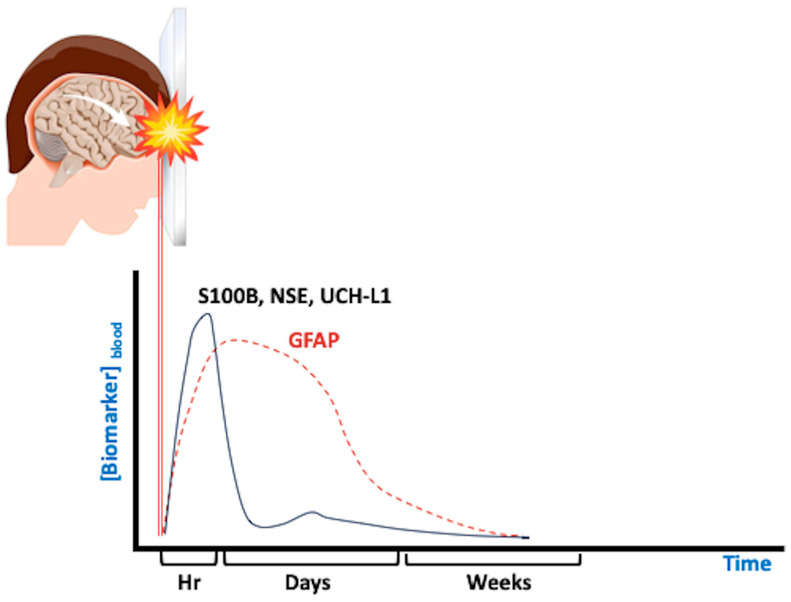
Different phases of traumatic brain injury can be monitored by a range of serum biomarkers: in the figure are some of the most studied ones. Modified from ref. [51]. S100B: calcium-binding protein B; NSE: neuron-specific enolase; UCHL1: ubiquitin carboxyl-terminal hydrolase isozyme L1; GFAP: glial fibrillary acidic protein.

**Scheme 1:**
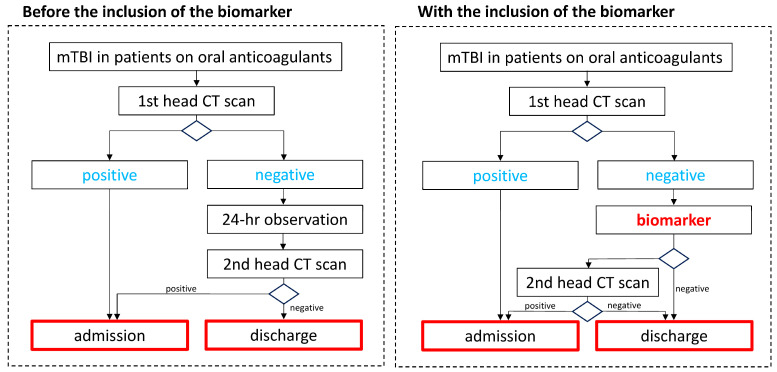
Hypothesis of new management of minor head injury based on the use of biomarkers in anticoagulated patients admitted to emergency departments. MHI: minor head injury; CT: computed tomography.

*Take home message*: In anticoagulated patients with mTBI brain damage, plasma biomarkers, such as S100B and GFAP, appear to be promising in predicting dICI during the observation period after a first normal CT scan (Figure 1, Figure 2 and Table 1). This strategy could reduce unnecessary resource wasting, without missing dICI, but these findings are worth future dedicated trials (Scheme 1).

## 4. Management: Not Only Neurosurgery

### 4.1. Setting: Intensive Care Unit/Neurosurgery/Emergency Department

Community health services should refer people who sustain a head injury to a hospital ED, particularly if they have risk factors including current anticoagulant or antiplatelet treatment (except aspirin monotherapy) [21]. Patients who sustain a TBI must be transported directly to a major trauma center that has the appropriate resources to resuscitate them and manage multiple injuries. For patients with a GCS score of 8 or less, the early involvement of an appropriately trained clinician is necessary to provide advanced airway management, especially for those who need to transfer to a neuroscience unit. Transfer would benefit anyone with sTBI, regardless of the need for neurosurgery. The purpose of the BIG project (see also above) was to define the best therapeutic management for three categories of patients, based on history, physical examination, and CT scan findings [22]. Patients on antiplatelet or anticoagulant medications were classified in the third category of severe head injury (BIG 3). The optimal plan for these patients consisted of hospitalization, neurosurgical consultation, and repeated head CT scans. This was supported by the evidence that 21.6% of the patients in BIG 3 had worsened results on the second head CT scan, with subsequent neurosurgical intervention in 3% of them. Nevertheless, no study has so far confirmed the real need to put patients on OAT or antiplatelet therapy into the most severe category of the BIG protocol [61,62]. As described above, Gallagher et al. [24] proposed a modified BIG for TBI patients, in which the OAT/antiplatelet criterion was excluded from the third category. This study found that none of the patients in the first and second categories (BIG 1 and 2) required neurosurgical intervention, and the utilization of neurosurgical consultation decreased by 52%. The authors concluded that the modified criteria may offer an opportunity to decrease neurosurgery consultations in anticoagulated patients presenting m- to mo-TBI, also guaranteeing patients’ safety while reducing costs.

*Take home message*: Even in the absence of robust evidence, we think that the modified BIG for TBI patients proposed by Gallagher et al. could enable emergency physicians to better identify which anticoagulated patients suffering from m- to mo-TBI need the “full” plan consisting of hospitalization, neurosurgical consultation, and repeated head CT scans.

### 4.2. Observation and CT Repeating

The 2023 NICE GL recommends that a person with TBI should be urgently re-evaluated and have access to an urgent CT scan if there is any sign of neurological deterioration, such as agitation or abnormal behavior, a sustained drop in GCS score, severe or increasing headaches, persistent vomiting, and new or evolving neurological symptoms [21]. Conversely, this GL does not formulate specific recommendations regarding if and when a CT scan should be repeated after the first CT scan positive for ICI in patients in stable clinical conditions. Some studies found that a routine repeat head CT scan is not indicated unless a neurological deterioration becomes evident, while others suggest that routine repeating of imaging is necessary to identify the subset of patients without neurological deterioration who could nonetheless require neurosurgical intervention [63,64]. Brown et al. [65] prospectively evaluated 163 patients with an initial abnormal head CT scan and found that CT scans triggered by neurological changes (19% of the patients) led to surgical intervention in 38% of cases, whereas scans obtained without any neurological alteration led to an intervention in only two patients (1%), both with a GCS score ≤ 8. Similar conclusions were found by Connon et al. [66]. Although there is no robust evidence on the timing for repeating brain CT scans, most authors agree that an interval between 6 and 24 h after the injury could be considered adequate [67]. Any eventual indication for repeating CT scans in anticoagulated patients with TBI regardless of the result of the first scan is even more conflicting. Despite this, there seems to be enough consensus on repeating CT scans between 6 and 24 h from TBI [23]. 

*Take home message*: Identifying factors associated with the increased risk of ICI progression, especially in patients on OAT, is a high-priority area for future clinical research.

### 4.3. Assessment of the Extent of Anticoagulation

The extent of ongoing anticoagulation warranted by AVKs is easily monitored by widely available blood tests, such as prothrombin time (PT) or the International Normalized Ratio (INR) [68]. The use of DOAs in daily clinical practice does not require the monitoring of coagulation, but the assessment of their anticoagulant effect could be desirable in critical situations, such as TBI. When interpreting a coagulation assay in a patient treated with a DOA, it is important to know how much time has elapsed between the blood draw and the last drug intake. No study has investigated the relationship between drug levels or the need for dose adjustment and the results of coagulation tests. Moreover, routine tests, such as PT, activated partial thromboplastin time (aPTT), and activated clotting time do not provide an accurate assessment of DOA anticoagulant effects and should not be used to evaluate anticoagulant activity [23]. However, these tests can provide some information. A normal aPTT excludes supratherapeutic levels in dabigatran-treated patients. Also, the effect of apixaban, edoxaban, and rivaroxaban can prolong PT, depending on the type of reagent used. Therefore, a normal PT does not exclude therapeutic levels [69]. In clinical trials, DOA plasma levels were measured using high-performance liquid chromatography/mass spectrometry, and specific assays for DOAs are available and in regular use nowadays [70]. Drug activity can be closely approximated using a calibrated ecarin chromogenic assay (ECA) and the diluted thrombin time (dTT) test for dabigatran or chromogenic anti-FXa assay [71]. The absence of anti-Xa activity excludes clinically relevant drug levels. However, clinicians must ask for specific assays for apixaban, rivaroxaban, or edoxaban. At the same time, the dTT test and the ECA display a direct linear relationship with dabigatran concentrations. An anti-Xa activity or dTT < 30 ng/mL excludes remaining DOA-associated anticoagulation. Point-of-care tests are also available, even if not yet widely used [72]. Urine tests may be useful for detecting exposure to DOAs, but levels do not strictly correlate with plasma concentrations [73]. The possibility of using viscoelastic tests is an intriguing future option in the management of hemorrhagic complications of DOAs, as they can quickly provide useful information. Unfortunately, there are not enough data supporting the use of thromboelastography or rotational thromboelastometry for a reliable assessment of DOA activity so far [73]. However, there is some evidence that assays, such as ClotPro assays, could have high levels of sensitivity and specificity for detecting clinically relevant drug levels of DOAs [73].

*Take home message*: In cases of TBI in a patient on therapy with DOAs, if there is doubt about the timing of the last dose of drug taken, drug activity can be closely approximated using ECA and dTT tests for dabigatran or chromogenic anti-FXa assays for the other DOAs. Viscoelastic tests appear promising, but little evidence is currently available.

### 4.4. Reversing Anticoagulation

In hemorrhagic patients treated with VKAs, vitamin K supplementation and the administration of four-factor prothrombin complex concentrates (4F-PCCs) rapidly restore INR values, with a low risk of volume overload [74]. For DOA-related bleeding, non-specific reversal agents such as fresh frozen plasma (FFP) and 4F-PCCs were the only possible strategies [74] until 2015, when the FDA approved the first specific reversal agent idarucizumab, a specific antidote for the thrombin inhibitor dabigatran [75]. In 2018, andexanet alfa was demonstrated as effective in reversing factor Xa inhibitor (FXaIs) classes [76]. Finally, ciraparantag emerged as a universal reversal agent, still under clinical development, designed to reverse both direct thrombin inhibitors and FXaIs, as well as the indirect inhibitors enoxaparin and unfractionated heparin (UFH) [74]. The use of these agents could be useful in effectively reducing the risk of bleeding in conditions such as TBI. The most appropriate positioning of DOA reversal therapy in the event of TBI is debated. In a recent consensus statement [23], authors affirmed that there is insufficient evidence to recommend DOA reversal in all patients with TBI. Expert opinion, based on clinical practice, limits DOA reversal in the case of a positive head CT scan and GCS < 14. The risks associated with rapid anticoagulation reversal, the eventual presence of hepatic and/or renal dysfunction, and costs should be carefully weighed against the potential benefits. In another consensus document, Iaccarino et al. [77] suggested stratifying anticoagulated patients with TBI based on low or high risk for bleeding and thrombosis, before making the decision to use reversals.

#### 4.4.1. Idarucizumab: Antidote for Direct Thrombin Inhibitor

Idarucizumab (aDabi-Fab, BI 655075) is a humanized murine monoclonal antibody fragment that matches both free and thrombin-bound dabigatran; the bound complex is eliminated primarily through the kidneys [75,76,77]. Dabigatran has nearly 350 times higher affinity for this antidote than for thrombin. Idarucizumab is administered by intravenous infusion in two boluses of 2.5–5 mL within 15 min, and its half-life is 45 min [75]. In comparison to healthy volunteers, the AUC concentration of idarucizumab rises from 43.5% to 83.5% in subjects with mild and moderate renal failure, respectively, while age, body weight, sex, and ethnicity have no relevant effect on pharmacokinetics [78]. An assessment of dabigatran concentration is not required before or after idarucizumab administration, nor is a check of the reversal effect on anticoagulant activity (see above) [79,80]. Headache, nasopharyngitis, back pain, and skin irritation are the most frequently reported adverse events, while no prothrombotic complications were described in the trials [75,78]. The RE-VERSE ADTM (study of the REVERSal of Effects of Idarucizumab in Patients on Active Dabigatran) [81], a multicentric, registrative, open-label study, showed a 30-day mortality rate of 18.79%, while thrombotic events occurred in 6.3% of patients treated with reversal therapy; no serious accidents attributable to the drug were reported. A recent SR confirmed these findings [82].

Only two case reports support the use of idarucizumab in TBI patients on dabigatran: the two patients, suffering from traumatic subdural hematoma, received idarucizumab before craniotomy without any further extension of the subdural hematoma [83,84]. More recently, Suehiro et al. [85] reported 23 cases of sTBI (mean GCS of 8.7) treated with idarucizumab; worsening levels of consciousness were observed in 30.4% of all patients, but only in 13.3% of subjects treated quickly with idarucizumab. In 13.1%, there were ischemic complications, all occurring beyond 7 days after the administration of idarucizumab.

#### 4.4.2. Andexanet Alfa: Universal Antidote for Factor Xa Inhibitors

Andexanet alfa (PRT064445) is a recombinant and inactivated form of FXadecoy protein. This class-specific antidote mimics FXa, without the direct catalytic activity of the original protein, due to a mutation in the protease complex. Andexanet alfa binds with high-affinity apixaban, rivaroxaban, and edoxaban, as well as low-molecular-weight heparin (LMWH) and fondaparinux-activated antithrombin III [76]. Andexanet alfa has a half-life of 1 h; thus, it has to be initially administered as a bolus (400–800 mg over 15–30 min), followed by a 2 h infusion of 2 to 2.5 h of 480–960 mg. The approval of andexanet alfa was supported by the ANNEXA-4 study (Andexanet Alfa, a Novel Antidote to the Anticoagulation Effects of FXA Inhibitors) [86], an open-label study without a control group, to allow for a comparison with “standard care” without antidote availability. In 80% of patients, excellent or good hemostasis was observed after 12 h; arterial or venous thrombotic events occurred in 12 of 67 patients (18%) during the 30-day follow-up. The mortality rate following ICI in patients of the ANNEXA-4 study treated with andexanet alfa was 21.4% (6 deaths in 28 patients with ICI). A recent SR confirmed these findings [82].

Only case series support the use of andexanet alfa in TBI patients on rivaroxaban or apixaban. One case of the successful rescue use of andexanet alfa was described by Maragkos et al. [87]. In a retrospective study, Sadek et al. [88] evaluated the outcome of reversal with andexanet alfa (59 patients) and 4F-PCCs in patients with isolated TBI (Abbreviated Injury Scale > 2 for head and <3 outside of head). They did not find any difference in mortality or severe hospital complications (28.5% and 8.5%, respectively, in andexanet alfa and 4F-PCCs group).

### 4.5. Tranexamic Acid

The Clinical Randomisation of an Antifibrinolytic in Significant Haemorrhage-2 (CRASH-2) trial demonstrated a significant reduction in mortality and deaths related to bleeding in patients treated with tranexamic acid (TXA) in the setting of polytrauma [89]. Afterwards, the CRASH-3 trial [90], an international, multicenter, randomized, double-blinding, placebo-controlled trial, investigated the use of TXA in the context of TBI and a GCS ≤ 12 or any intracranial bleeding on a CT scan. The dosage used in this study was 1 g over 10 min (loading dose), followed by an infusion of 1 g over 8 h. In this study, investigators claimed that TXA is safe and can reduce the risk of head injury-related death when administered within 3 h of injury to patients with mTBI or moTBI, but not in the population with seTBI. The risk of vascular occlusive events was similar in the placebo group. However, these trials do not mention eventual ongoing anticoagulant treatment. TXA was tested for treating acute ICI, regardless of the cause (also traumatic), in several RCTs, such as TICH-2 (Tranexamic Acid for Hyperacute Primary IntraCerebral Haemorrhage) [91], STOP-AUST (Tranexamic Acid in Patients with Intracerebral Haemorrhage) [92], and TRAIGE (Tranexamic Acid for Acute Intracerebral Haemorrhage Growth based on Imaging Assessment) [93], but these trials excluded patients with DOA-associated ICI. TICH-NOAC (Tranexamic Acid for Intracerebral Hemorrhage in Patients on Non-Vitamin K Antagonist Oral Anticoagulants) [94] was a multicenter, randomized, placebo-controlled trial for patients with DOA-associated ICI. This study showed no evidence that TXA prevents hematoma expansion or improves clinical outcomes. Thus, it appears correct to affirm that there is no evidence that TXA improves outcomes in anticoagulated patients suffering TBI so far [23]. However, the European GL on the management of major bleeding and coagulopathy following trauma [95] states that the co-administration of TXA is indicated for trauma patients independently of OAT and the adoption of a reversal strategy. Once again, however, this is a general recommendation for trauma, not one specifically addressing the case of TBI. A very recent retrospective study [96] collected trauma patients with pre-existing anticoagulation (of whom only 20.8% were on DOAs and 21.9% were on VKAs) from the TraumaRegister DGU^®^. Of them, 996 (17.2%) received TXA with a positive effect on 24 h mortality (OR 0.77; 95% CI: 0.61–0.98; *p* = 0.05). The authors did not find a relevant difference in thromboembolic complications: 3.3% vs. 3.1%, respectively, in the TXA and no TXA groups (*p* = 0.823).

**Figure 3:**
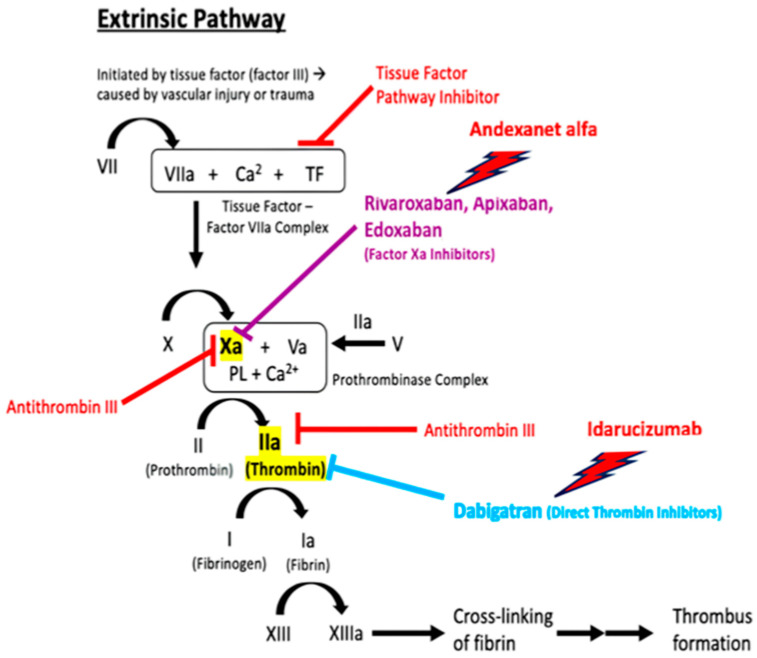
Site of anticoagulants and reversal agents in the coagulation cascade. Modified from ref. [95].

*Take home message*: Actual standard therapy for ICI after TBI in anticoagulated patients consists of the administration of TXA, if not yet in progress, together with the use of 4F-PCCs and specific reversal in cases of VKA and DOAs, respectively (Figure 3). However, benefits of the use of andexanet alfa should be carefully weighed against its costs, possibly relying on an interdisciplinary discussion. Moreover, it is notable that andexanet alfa is not yet licensed for edoxaban reversal. 

### 4.6. Indications for Neurosurgery

Evidence about indications for neurosurgery after TBI in patients on OAT is scarce and inconsistent, hampering the formulation of reliable recommendations for clinical practice. Regardless of the use of anticoagulants, the NICE GL recommends discussing together with a neurosurgeon the care of patients with significant neuroimaging alterations, persisting coma after initial resuscitation, unexplained confusion that persists for more than 4 h, deterioration in GCS score after admission, progressive focal neurological signs, seizures without full recovery, definite or suspected penetrating injuries, and cerebrospinal fluid leak [26]. The last Brain Trauma Foundation GL for the management of sTBI [97] provided recommendations about decompressive craniectomy and cerebrospinal fluid drainage, but no specific considerations for patients on OAT. In the case of acute subdural hematoma (aSDH), the US GL states that acute SDH > 10 mm in thickness or with a midline shift > 5 mm on a CT scan should be surgically evacuated, regardless of the patient’s GCS score [98]. On the other hand, a 2021 meta-analysis showed high rates of mortality (49% at long-term follow-up) and poor neurological outcomes in patients > 65 years old following surgery for aSDH [99]. Frequently, older patients with aSDH are observed for 14 days in order to monitor the evolution of aSDH to the chronic type. For symptomatic chronic SDH, the less invasive burr-hole drainage is considered the first-line surgical intervention [100]. Asymptomatic chronic SDH, instead, should be managed conservatively, because the risks exceed the benefits of surgery. Middle meningeal artery embolization (MMAE) is a new, minimally invasive, and encouraging treatment for chronic and acute-on-chronic SDH performed by interventional radiology. A retrospective study that included adult patients with isolated TBI on anticoagulants showed that there was a very low (0.023%) prevalence of dICI and, consequently, a very low prevalence of neurosurgery [101]. Another retrospective study reported that in anticoagulated patients suffering traumatic aSDH, the use of anticoagulant reversal therapy may reduce the risk of hemorrhagic complications and mortality after neurosurgery [102]. A recent SR [62] found that in the case of ICI after mTBI, patients using DOAs received reversal agents as a bridge to neurosurgery less often compared to patients using VKAs. This was because most of the included studies were conducted before the approval of new reversals. Moreover, in clinical practice, when considering a reversal agent, physicians assess the likelihood that the anticoagulant contributes to progressive bleeding, and the measurement of the anticoagulant activity of DOAs is more complex than the anticoagulant activity of VKAs.

*Take home message*: Indications for neurosurgery in patients on OAT are the same for those in patients not on OAT. In the case of mTBI, data showed a significantly lower rate of neurosurgical intervention in patients using DOAs compared to patients using VKAs. 

### 4.7. Prophylaxis and Anticoagulant Resumption after TBI

When TBI occurs in patients taking OAT, the medication should be discontinued to prevent hematoma expansion. On the other hand, these patients are also at significant risk of thromboembolic events [103]. The efficacy of pharmacological prophylaxis in preventing thromboembolic events after TBI is well established in the updated Brain Trauma Foundation guideline [67]. In a recent SR [104] and GL [105], the authors concluded that low-molecular-weight heparin (LMWH) or UFH can be safely administered as early as 24–48 h post-injury for patients with low-hemorrhagic-risk TBI and stable pictures upon repeated imaging. However, TBI patients receiving OAT were excluded from the selected studies. A large retrospective study of trauma patients demonstrated that LMWH performed better than UFH, but, again, it is unclear whether this finding is applicable to TBI patients on OAT.

**Figure 4:**
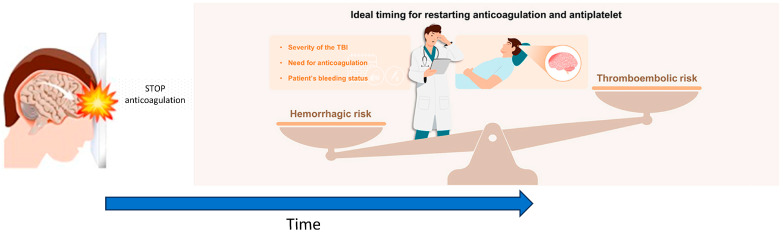
Steps in the management of anticoagulation after traumatic brain injury. Modified from ref. [103].

After hemostasis is achieved and traumatic hemorrhage has stopped, a conflict between the need for the resumption of the antithrombotic agent due to the underlying disease and the risk of progression or recurrence of the intracranial hemorrhage emerges (Figure 4). Nielsen et al. [106] found that anticoagulant resumption appears to be associated with ICI recurrence in patients with hemorrhagic stroke, but this association has not been confirmed in patients with TBI (adjusted hazard ratio: 0.45; 95% CI: 0.26–0.76). In a retrospective study of 10,782 patients on warfarin, Albrecht et al. [107] reported that there was a net benefit of warfarin resumption after TBI because it decreased the combined risk of hemorrhagic or ischemic stroke (RR: 0.83; 95% CI: 0.72–0.96). The optimal timing for the resumption of the antithrombotic agent remains controversial, too. Before restarting anticoagulants, several factors should be considered, including the severity of the TBI, the patient’s bleeding status, and the real need for anticoagulation. Puckett et al. [108] and Naylor et al. [109] conducted retrospective studies on patients with TBI, claiming that adverse events were minimal when therapy was restarted 7–9.5 days and 30 days after the injury, respectively. In a multicenter, prospective, observational study, Matsushima et al. [110] found that the re-initiation of OAT within 30 days after TBI was associated with a higher risk of TBI progression, too. In a recent GL [23], it was recommended that the decision to resume OAT should be made on a case-by-case basis. However, according to international GLs for the management of spontaneous ICH, the authors suggested that therapeutic anticoagulation can be restarted 10–14 days after TBI in patients with a stable injury at a high risk of thrombotic complications (i.e., those with mechanical valve prosthesis or non-valvular atrial fibrillation and a CHA2DS2VASc score ≥ 4 or antiphospholipid syndrome with recurrent thromboembolic events). In patients with a moderate or low risk of thromboembolic events, it may be more appropriate to resume anticoagulation 4–8 weeks after TBI [23]. A recently published consensus document recommended that some interventions, such as stopping VKA with the use of 4F-PCCs or stopping DOAs with or without reversal, should be chosen based on the risk stratification of the patients with low or high bleeding/thrombosis risk [77].

An even more challenging situation is the concomitant presence of TBI and active thromboembolic disease. Chipman et al. [111] did not find a significant difference in the progression of post-traumatic ICI in 50 patients with concomitant pulmonary embolism between those who started anticoagulation therapy within 7 days from injury and those who started it after 7 days from injury. Byrnes et al. [112] reported 42 patients with traumatic ICI who subsequently developed thrombotic complications; ICI remained stable in 25 out of 26 patients who started therapeutic anticoagulation 13 days after the injury and bleeding signs slightly increased in only one patient. As an alternative, there are reports suggesting the use of inferior vena cava filter insertion together with pharmacological prophylaxis [113].

The decision about whether and when resuming therapeutic antithrombotic therapies after the acute phase needs a case-by-case evaluation; however, OAT can be safely restarted 4 weeks after TBI in the absence of a high risk of thrombotic complications, such as mechanical valve prosthesis.

*Take home message*: The decision about whether and when to resume therapeutic antithrombotic therapies after the acute phase needs a case-by-case evaluation; however, OAT can be safely restarted 4 weeks after TBI in the absence of a high risk of thrombotic complications, such as mechanical valve prosthesis.

## 5. Complications

### 5.1. Delayed Intracranial Injuries

Some ICIs are not evident at the first scan after TBI, especially when mTBIs occur, and this event is denoted as “delayed ICIs” (dICI, see above). The definition varies, but usually ranges from an intracranial hemorrhage found within 24 h of trauma to 2 to 30 days [114]. Data about the frequency of dICI following an mTBI are very uncertain, ranging from 0.5 to 2% reported in some studies to 9 to 13% reported in others [35,39,41,61,115,116,117,118,119].

These differences are, in part, consequences of methodological issues, such as an overall poor quality of the studies, mostly characterized by their retrospective design and affected by a high prevalence of missing or unclear data. Moreover, most of the studies enrolled mixed populations treated with different types of antithrombotic and antiaggregant therapies [120,121], while others enrolled only patients on DOAs [119,122]. Of course, this heterogeneity undermines the reliability of most of the SRs [35,39,115,116,117]. More recently, two SRs [116,117] found only two prospective studies investigating exclusively patients on VKAs or DOAs [118,123]. In the cohort of Cohan [118], the dICI incidence was 2.3% in the DOA group and 4% in the VKA group (*p* = 0.31). Two VKA patients received neurosurgical intervention, and three died from their TBI, while none needed neurosurgery or died in the DOA group. In the study by Cipriano et al. [122], 3 out of 178 anticoagulated patients showed a dICI (1.7%; 95% CI: 0.0–3.6%), and 1 of them died (0.6%; 95% CI: 0.5–1.7%), while the others did not require neurosurgical intervention. In two independent prospective cohorts [11,42], we found an incidence of dICI of 6% and 4.7%, respectively. In the former study [11], 3 patients were subsequently hospitalized and 1 received craniotomy, while, in the latter [42], 17 patients were admitted and no neurosurgery was needed and no death occurred.

To avoid missing dICIs, current GLs or protocols recommend observation and rescanning. However, because serious complications appear to be rare, observing and rescanning all anticoagulated patients is controversial. The rate of incidence of adverse events could be judged to be too low to warrant the costs, inconvenience, and risks associated with further observation and repeated imaging. On the other hand, establishing any “acceptable” risk threshold is very sensitive to values provided by the various stakeholders (patients, clinicians, managers), depending on factors such as fear of clinical consequences, fear of litigation, organizational burden, and costs. So, the risk stratification of mTBI patients on OAT appears to be very important in selecting the group of patients who may benefit from a repeated head CT scan [124]. 

*Take home message*: We propose using one or more biomarkers (Scheme 1) in combination with clinical predictors (see above, Section 3.2) to better select which patients should be observed and undergo a second head CT scan or be safely discharged.

### 5.2. TBI-Induced Coagulopathy

In TBI, the primary injury is due to the direct impact on the brain, which causes hemorrhagic lesions through cerebral blood vessel disruptions or contusional lesions to the brain parenchyma [125]. About one-third of patients with moTBI to sTBI develop a coagulopathy within the first 24 h, called TBI-induced coagulopathy (TBI-IC), associated with an augmented risk of hemorrhagic progression, poor neurological outcomes, and death [126]. Direct vessel injury or defragmentation from microvascular failure and tissue hypoperfusion trigger the release of tissue factors, lead to the excessive production of thrombin, and cause a massive release of the tissue-type plasminogen activator (t-PA) and urokinase-type plasminogen activator (u-PA), which activate plasmin and lead to secondary fibrinolysis [127]. This state of hyperfibrinolysis could contribute to the bleeding diathesis [128]. Low platelet counts and platelet dysfunction seem to be another component of TBI-IC. The diagnostic criteria of TBI-IC are a PT-INR > 1.1–1.5, aPTT > 32–60 s, platelet count < 50–120 × 10^9^/L, fibrinogen concentration < 1.5–2.0 g/L, D-dimer elevation, and α2-plasmin inhibitor (α2-PI) levels < 60% of normal value [127]. Viscoelastic tests, such as rotational thromboelastometry (ROTEM) and thromboelastography (TEG), seem to be more specific and make the earlier detection of TBI-IC possible, enabling goal-directed therapy, even if only low-quality evidence is available [129,130].

There are several independent risk factors for TBI-IC, such as hypotension, hypothermia, hypoxia, and reduced GCS [126]. The pre-injury intake of anticoagulants seems to correlate with a higher incidence of TBI-IC. In the recent CENTER-TBI observational study [131], TBI-IC was found in 16% of patients with isolated TBI and no pre-injury intake of anticoagulants versus 34% of patients taking OAT. The pathophysiology behind the coagulopathy is not completely clear [125]. Therapeutic thresholds remain poorly defined, too. Currently, a standard therapy for TBI-IC has been adopted from that of trauma-induced coagulopathy and involves administering TXA, FFP, or 4F-PCCs; supplemental fibrinogen and calcium; and packed red blood cell concentrates [129]. Goal-directed therapies by a viscoelastic assay-guided algorithm to restore hemostasis in TBI patients were proposed [132], but further studies are needed.

*Take home message*: The assessment of the extent of anticoagulation could be useful, at least in the most severe cases, and viscoelastic tests could be effective for this purpose, allowing at the same time the rapid identification of variation of the coagulable state, such as TBI-IC and hyperfibrinolysis. 

## 6. The Economic Burden

TBI has a massive economic impact on individuals, families, and the entire society. A thorough analysis of TBI health economics could be enhanced to develop more efficient care and prevention. However, available information on TBI costs is still scarce and controversial, with large differences among the studies, related to discrepancies in methods used to estimate costs and even to variability in the definitions of direct, indirect, and lifetime costs [133,134]. One of the first important epidemiological sources of reliable data about the incidence of TBI, stratified by age classes and severity score, is the BIONIC (Brain Injury Outcomes New Zealand In the Community) study [135]. In this analysis, although every single case of mTBI was burdened by lower costs, the high absolute and relative prevalence of mTBI led to a cumulative cost almost three times higher than that due to moTBI and sTBI together. BIONIC and other studies did not evaluate OAT exposition as a specific cost predictor [136]. Choksi et al. [137] reported that the odds of inpatient complications and costs increased in the presence of comorbidities requiring the use of OAT. Using a decision-analysis model, Kuczawski et al. [138] suggested that head CT scans for all anticoagulated patients with TBI are not cost-effective since they produce an incremental cost of GBP 94,895 per quality-adjusted life year (QALY) gained, which is greatly above the usual threshold of GBP 20–30,000/QALY for a fair cost-effectiveness that NICE adopts.

Reversal therapies are definitely expensive, in particular andexanet alfa. However, the absence of head-to-head trials comparing idarucizumb or andexanet alfa vs. 4F-PCCs after TBI does not make it possible to draw conclusions about their potential cost-effectiveness. Indeed, there are only a few published economic analyses on the use of those specific reversals [139,140]. In particular, Fakinos et al. [140] affirmed that the use of andexanet alfa for the reversal of anticoagulation in patients with FXaI-related ICI is likely to be cost-effective.

*Take home message*: Only scarce and conflicting results are currently available, in particular for patients exposed to OAT, making this one of the hottest topics for future research.

## 7. Future Directions: The Role of Artificial Intelligence-Assisted Decision

Artificial intelligence (AI) has the ability to improve the accuracy and speed of interpreting large datasets comprising images, speech, and text [141]. Machine learning (ML) could estimate outcomes, based on the combination of past experiences and emerging data analysis, using computer-generated algorithms. Many ML models are currently under evaluation in medicine, particularly for injured patients: artificial neural network, singular vector machine, Bayesian network, random forest, natural language processing, stacked ensemble classifier, SuperLearner (SL), k-nearest neighbor, belief system, and sequential minimal optimization models [142]. 

In mTBI, ML can assist in the screening of patients for whom a head CT scan should be recommended [143]. For example, the quantitative interpretations of electroencephalography signal (qEEG)-based techniques appear promising in detecting mTBI cases [142,143]. However, patients on OAT have always been excluded from these studies so far.

In moTBI, ML proved promising in predicting patient outcomes and determining the likelihood of deterioration or the need for intervention [144,145]. However, again, patients on OAT were excluded from the analysis. In sTBI, the fields of interest for AI are the classification of ICI, the prediction of increased intracranial pressure (ICP) or ICP estimation, midline shift (MLS) detection/quantification, and TBI prognostication [144]. However, OAT was again an exclusion criterion in all published studies. In the work of Tu et al. [146], a logistical regression (LR)-based model was the best model (AUC of 0.925) for mortality risk prediction of patients with TBI in the emergency room triage. The external validation on 200 patients revealed that this study’s model is acceptably stable and reliable for helping physicians’ decision-making. 

Of course, several conditions still need to be met before the widespread implementation of ML techniques can be recommended, such as a large availability of big datasets together with the on-site availability of effective and safe technical requirements.

*Take home message*: The introduction of AI as a clinical decision support tool in this field of emergency medicine is highly promising and, thus, further studies in a real-world setting are urgently needed.

## 8. Conclusions: A New Classic Topic with New Answers

Several very relevant issues still remain immersed in the fog of scarcity of robust evidence coming from clinical research. Some of these are definitely characterized by the highest priority: (a) a univocal definition of mTBI, (b) the best cost-effective indication for a second head CT scan in the case of mTBI, (c) the timing of a repeated head CT scan in the case of moTBI or after a diagnosis of ICI in a stable patient, (d) a flow chart about the use of reversals and TXA in mo/sTBI, and (e) the most appropriate follow-up after an mTBI.

## Figures and Tables

**Table 1:** Testing brain biomarkers after mTBI in patients on oral anticoagulants. S100B: calcium-binding protein B; NSE: neuron-specific enolase; UCHL1: ubiquitin carboxyl-terminal hydrolase isozyme L1; GFAP: glial fibrillary acidic protein.

Biomarker	Study	Patients on OAT Included	Analysis of the Patients on OAT	Results	Reference
S100B	Biberthaler et al. 2006	0/1309	No	/	[47]
	Laribi et al. 2014	0/431	No	/	[48]
	Morochovič et al. 2009	0/102	No	/	[49]
	David et al. 2017	83/308	No	NPV 94.3% (95% CI: 87.2–98.1%) PPV 12.7% (95% CI: 8.6–17.9%)	[54]
	Menditto et al.	313/313	Yes	NPV 86.9% (95% CI: 82.5–90.3%) PPV 15.9% (95% CI: 12.1–20.5%)	[55]
NSE	Menditto et al.	313/313	Yes	NPV 84.3% (95% CI: 79.7–88.1%) PPV 13.0% (95% CI: 9.6–17.4%)	[55]
UCHL1	Menditto et al.	313/313	Yes	NPV 88.1% (95% CI: 82.8–91.9%) PPV 17.3% (95% CI: 12.6–23.1%)	[55]
GFAP	Menditto et al.	313/313	Yes	NPV 95.8% (95% CI: 92.0–97.9%) PPV 16.6% (95% CI: 12.0–22.4%)	[55]

## Supplementary Materials

The following supporting information can be downloaded at https://www.mdpi.com/article/10.3390/jcm13133669/s1. Figure S1. Flow diagram of the study selection process.
