# Supplementary material for: Traumatic Brain Injury in Patients under Anticoagulant Therapy: Review of Management in Emergency Department
Source: J Clin Med. 2024 Jun 24;13(13):3669. doi: 10.3390/jcm13133669 (PMC11242576; doi:10.3390/jcm13133669)
Supplement: Supplementary file 1 [file jcm-13-03669-s001.zip › jcm-3008403-supplementary.pdf]

Supplementary file

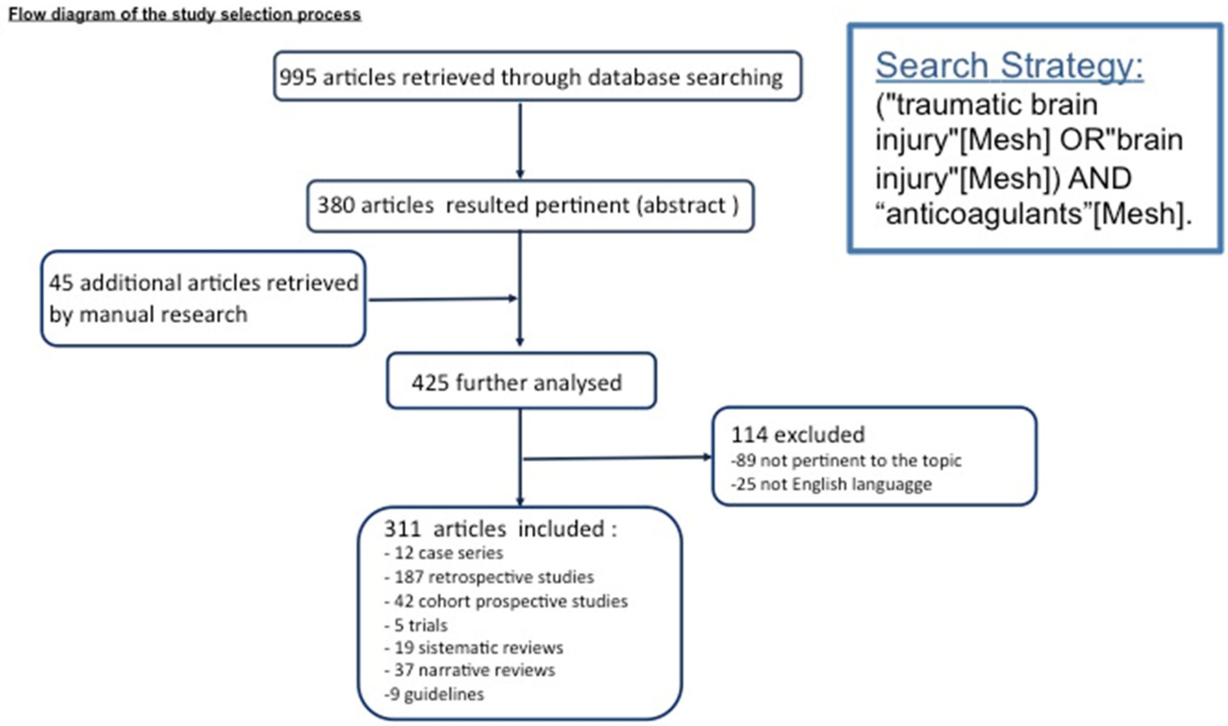

Figure S1. Flow diagram of the study selection process.
